# Improving the Tensile and Tear Properties of Thermoplastic Starch/Dolomite Biocomposite Film through Sonication Process

**DOI:** 10.3390/polym13020274

**Published:** 2021-01-15

**Authors:** Azlin Fazlina Osman, Lilian Siah, Awad A. Alrashdi, Anwar Ul-Hamid, Ismail Ibrahim

**Affiliations:** 1 Faculty of Chemical Engineering Technology, Universiti Malaysia Perlis (UniMAP), Arau 02600, Malaysia; msliiian0820@gmail.com (L.S.); ismailibrahim@unimap.edu.my (I.I.); 2 Biomedical and Nanotechnology Research Group, Center of Excellence Geopolymer and Green Technology (CEGeoGTech), Universiti Malaysia Perlis (UniMAP), Arau 02600, Malaysia; 3 Chemistry Department, Umm Al-Qura University, Al-Qunfudah University College, Al-Qunfudah Center for Scientific Research (QCSR), Al Qunfudah 21962, Saudi Arabia; aarashdi@uqu.edu.sa; 4 Center for Engineering Research, Research Institute, King Fahd University of Petroleum & Minerals, Dhahran 31261, Saudi Arabia; anwar@kfupm.edu.sa

**Keywords:** thermoplastic starch, dolomite, biocomposite, mechanical properties, sonication

## Abstract

In this work, dolomite filler was introduced into thermoplastic starch (TPS) matrix to form TPS-dolomite (TPS-DOL) biocomposites. TPS-DOL biocomposites were prepared at different dolomite loadings (1 wt%, 2 wt%, 3 wt%, 4 wt% and 5 wt%) and by using two different forms of dolomite (pristine (DOL(P) and sonicated dolomite (DOL(U)) via the solvent casting technique. The effects of dolomite loading and sonication process on the mechanical properties of the TPS-DOL biocomposites were analyzed using tensile and tear tests. The chemistry aspect of the TPS-DOL biocomposites was analyzed using Fourier transform infrared spectroscopy (FTIR) and X-Ray Diffraction (XRD) analysis. According to the mechanical data, biocomposites with a high loading of dolomite (4 and 5 wt%) possess greater tensile and tear properties as compared to the biocomposites with a low loading of dolomite (1 and 2 wt%). Furthermore, it is also proved that the TPS-DOL(U) biocomposites have better mechanical properties when compared to the TPS-DOL(P) biocomposites. Reduction in the dolomite particle size upon the sonication process assisted in its dispersion and distribution throughout the TPS matrix. Thus, this led to the improvement of the tensile and tear properties of the biocomposite. Based on the findings, it is proven that the sonication process is a simple yet beneficial technique in the production of the TPS-dolomite biocomposites with improved tensile and tear properties for use as packaging film.

## 1. Introduction

Nowadays, petroleum-based plastics are daily and widely used packaging materials. Unfortunately, this contributes negative effects to the environment as a result of the long degradation time of plastics leading to waste disposal problems [1]. Scientifically, the molecular structure and chemical bonding of the petroleum-based plastic caused them to be too durable and resistant to the natural process of degradation. For example, polyolefins such as polypropylene (PP) and polyethylene (PE) are very resistant to hydrolysis and totally non-biodegradable. Consequently, this causes a large accumulation of plastic wastes in the landfill, causing a serious waste management problem and soil contamination. Moreover, the toxic chemicals present in the plastic will also cause pollution to the environment. When plastics are burned, they emit dioxin, which is the most toxic substance that may harm the health, reproduction, development, immune systems, disrupt hormones, cause cancer, and create other issues [2]. To reduce those effects, recycling of the plastic wastes is performed. However, several problems may arise during the recycling process, especially during the collection, separation, and cleaning, due to possible contamination on the plastics and difficulty of finding an economically viable outlet, where incineration may give off some toxic gas [3].

Due to the above-mentioned problems, effort to replace the synthetic plastics with the natural-based plastics is gaining more traction from year to year. The development of new plastic materials from natural and renewable resources has been the object of intensive academic and industrial research [4,5,6]. Starch-based biodegradable plastics are an ideal replacement to petroleum-based plastics. Even though these biopolymers cannot replace synthetic polymers in every application, at least they can be used to produce specific products, normally for application in which the recovery of plastic is not viable, economically feasible, and controllable such as in one-time use plastic [4,5,6,7].

Thermoplastic starch (TPS) can be obtained through plasticization of the native starch granules. TPS can be used to form biodegradable biocomposites films for packaging application. However, TPS-based products usually have several disadvantages. They may exhibit poor mechanical performance due to severe issues of water sensitivity, environmental stress cracking, and high fragility over time. Their low mechanical properties are a major concern, especially when exposed to hydrolytic and oxidative conditions and elevated temperatures [4,8]. These restrict the use of the TPS packaging films for long-term application as opposed to the petroleum-based plastic. In order to improve the properties, the TPS needs to be reinforced with fillers. In this research, a mineral filler, which is dolomite (DOL), was used to reinforce the TPS film. In this context, dolomite particles dispersion in the TPS matrix is very important, since it can affect the mechanical properties of the resultant TPS-DOL biocomposite films. Previous research indicated that a good dispersion of filler leads to improvement in the mechanical properties of the TPS composites, such as tensile and tear strength [4,8]. Generally, the pristine dolomite particles exist in agglomerated form. This may hinder their homogenous dispersion throughout the TPS matrix. The poor dispersion of dolomite can inhibit their reinforcing capability; thus, biocomposites films with poor properties may be produced [9]. Due to these reasons, dolomite needs to undergo a pre-dispersion process to diminish particle agglomeration, thus ensuring a good dispersion of dolomite in the TPS matrix. According to previous research, sonication is a practical method for the pre-dispersion process of the particles and nanoparticles [10]. Thus, this method was employed in this study to obtain a good dispersion of dolomite in the TPS matrix.

To the best of our knowledge, no research related to TPS/dolomite biocomposite film has been conducted and reported. Our research group is the first to study and investigate the TPS/dolomite biocomposite system for potential use as packaging film. Both starch and dolomite are abundant sources of materials to be transformed into biocomposite or other products with several beneficial applications. Thus, research in this area is considered impactful toward sustainability. Of particular interest, an optimization study on the TPS/dolomite biocomposite system can be the first step to formulate the biocomposite with the best property profiles. Therefore, the objective of this study was to investigate the effects of dolomite loading (0, 1, 2, 3, 4, and 5 wt%) on the tensile and tear properties of the TPS/dolomite biocomposite film. The novelty of this work is the use of a simple technique for pre-dispersing the dolomite, which is tip sonication process to improve dispersion of dolomite particles in the TPS matrix. This technique involved no harmful chemicals, as the medium used is only water. Furthermore, the application of this pre-dispersion technique allows the enhancement in both tensile and tear properties of the TPS biocomposite incorporating dolomite. This article highlights all of these aspects.

## 2. Materials and Chemicals

Corn starch powder, dolomite filler, glycerol, and sodium bicarbonate have been used to produce the virgin TPS film and TPS-DOL biocomposites film. Corn starch was manufactured by Sigma-Aldrich (St. Louise, MO, USA) with code number S4126 in 2 kg poly bottle packing. It was supplied by Euroscience Sdn. Bhd (Kuala Lumpur, Malaysia) in white powder form. It consists of about 27% amylose and 73% amylopectin. Dolomite was kindly supplied by Perlis Dolomites Industries Sdn. Bhd (Padang Besar, Malaysia) and is known by the locals as ‘batu reput’. It came in powder form with a particle size of 150 µ. Dolomite was used as filler in the TPS biocomposite film in pristine and sonicated form. Glycerol with a minimum concentration of 99.5% was manufactured by HmbG Chemicals (Hamburg, Germany) with code number C0347-91552409. This chemical was purchased through A.R. Alatan Sains Sdn Bhd (Alor Setar, Malaysia). Glycerol served as a plasticizer in the formulation of TPS film. Sodium bicarbonate was manufactured by HmbG Chemicals (Hamburg, Germany) with code number C0725-2134330 and supplied by A.R. Alatan Sains Sdn Bhd (Alor Setar, Malaysia). It acted as a thickener in the production of TPS film.

### 2.1. Preparation of Dolomite (DOL(P)) and Ultrasonicated Dolomite (DOL(U)

Dolomite powder having a particle size of 150 µ was ground into finer and smaller particle size by using a mortar and pestle. After that, it was sieved by using a 50 µ sieve with 100 mesh. The resultant powder is called pristine dolomite (DOL(P)).

The sonication of dolomite was done as a pre-dispersion process prior to biocomposite production to assist dolomite dispersion and distribution in the TPS matrix. The suspension of dolomite was prepared by mixing the powder form of dolomite with distilled water in a ratio of 1:100. The dolomite was sonicated by using a Branson Digital Ultrasonic Disrupter/Homogenizer, Model 450 D with 20% amplitude, 20 s pulse on, and 10 s pulse off for 120 min. The resultant sonicated dolomite is referred to as DOL(U).

### 2.2. Plasticization of Starch to Form Thermoplastic Starch (TPS)

Thermoplastic starch (TPS) was obtained through the combination of corn starch with plasticizers under a stirring and heating process. Firstly, distilled water, corn starch, and glycerol were combined in a mass ratio of 100:5:2 in one beaker. The amount of water, corn starch, and glycerol used to produce one film of TPS in a Teflon mold were 100 mL, 5 g, and 2 g, respectively. Next, the mixture was stirred continuously in a water bath under a temperature range of 75–85 °C by using a magnetic stirrer (500 rpm). This procedure took about 45 min or until the mixture turned into paste.

### 2.3. Preparation of TPS-DOL Biocomposites

**Figure 1:**
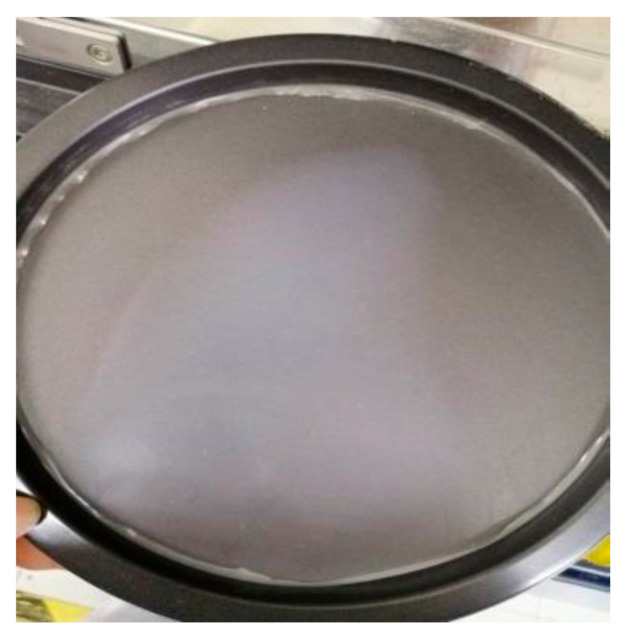
Dried film of the thermoplastic starch-dolomite (TPS-DOL) biocomposite in the Teflon-coated mould.

The preparation of the TPS-DOL biocomposites films was done through the solution casting and drying process method. The suspension of dolomite (sonicated) was added into the TPS suspension and keep stirred in the range between 75 and 85 °C. Next, 0.5 g of sodium bicarbonate was dissolved in 30 mL of distilled water and then added into the TPS suspension, which was continuously stirred for another 5 min. After that, the biocomposite suspension was poured into Teflon-coated mold and put into the oven at 45 °C for 24 h for drying. The dried film of the TPS-DOL biocomposite is shown in Figure 1. It was removed from the Teflon-coated mold and stored in a desiccator to avoid moisture while conditioning it in standard environment prior to testing. The process was repeated for pristine dolomite (without sonication) to form control biocomposites films. All the film samples were prepared in the same volume and using the same-size molds. Therefore, all samples had almost similar thickness.

The formulations of TPS-DOL biocomposite films are shown in Table 1. In preliminary works, we have prepared the TPS biocomposite films with dolomite loading greater than 5 wt% (7 and 9 wt%). However, no good films were observed, which was perhaps due to agglomeration and the overcrowding of dolomite particles in the matrix. The films were brittle and cracked after being cut into dumbbell shape. Furthermore, the samples took a longer time for drying. Based on these observations, we did not proceed for testing.

**Figure 2:**
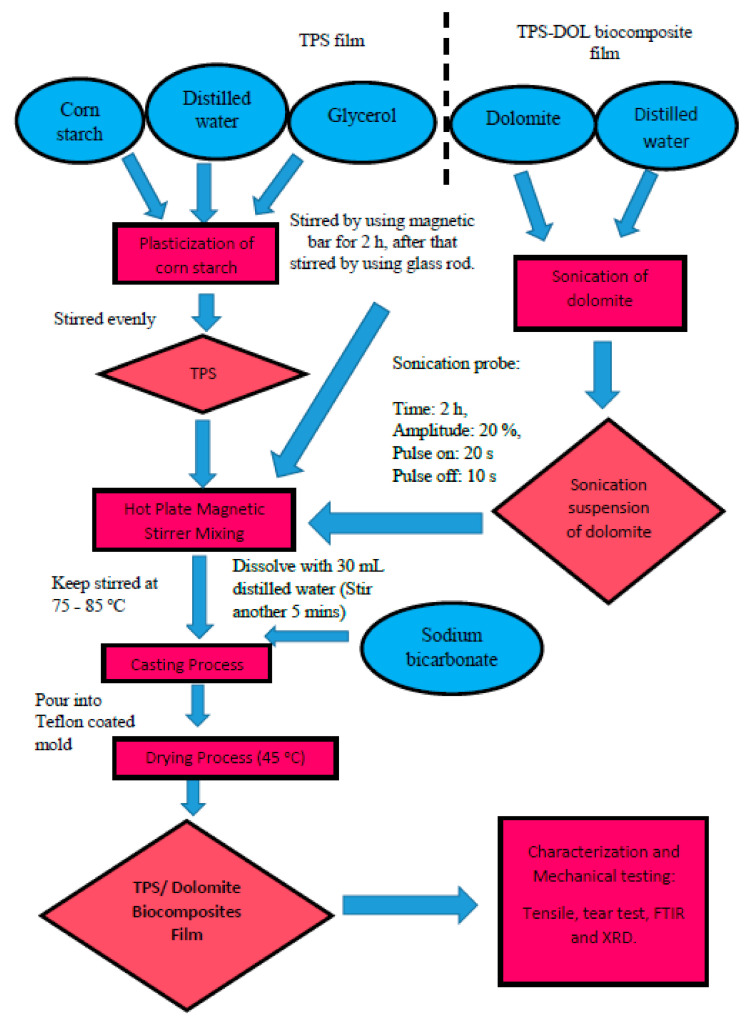
Flow chart of experimental procedures.

The overall experimental procedures to produce the TPS-DOL biocomposite films are summarized in Figure 2.

## 3. Characterization and Mechanical Testing

The tensile test, tear test, and FTIR analysis were conducted to test the properties and chemical structure of the TPS film and TPS-DOL biocomposite films.

### 3.1. Fourier Transform Infrared Spectroscopy (FTIR)

By using a Perkin Elmer RXI FTIR spectrophotometer (Waltham, MA, USA), the FTIR spectra of all the samples were analyzed. The chemical functional groups of TPS-DOL biocomposites (incorporating the DOL(P) and DOL(U)) were characterized by FTIR. The samples were recorded for 32 scans in the frequency range of 650–4000 cm^−1^ wavenumber with a resolution of 32 cm^−1^.

### 3.2. X-ray Diffraction (XRD)

X-ray diffraction (XRD) was used to analyze the structure of dolomite before and after the sonication process. The analysis was also performed on the TPS film and TPS-DOL biocomposite films incorporating the DOL(P) and DOL(U) to determine their crystallinity. Each sample was placed in sample holder and scanned over a range of 2θ = 10°–35° and with a scan speed of 2°/min.

The degree crystallinity of corn starch, TPS, DOL(P), DOL(U), and TPS-DOL biocomposite films was evaluated by using the formula below [11].

$$\mathrm{Degree}\;\mathrm{of}\;\mathrm{Crystallinity}=\frac{\left(\mathrm{total}\;\mathrm{area}\;\mathrm{of}\;\mathrm{cristalline}\;\mathrm{peaks}\right)}{\left(\mathrm{total}\;\mathrm{area}\;\mathrm{of}\;\mathrm{all}\;\mathrm{peaks}\right)}\times 100\%.$$

### 3.3. Tensile Test

**Figure 3:**
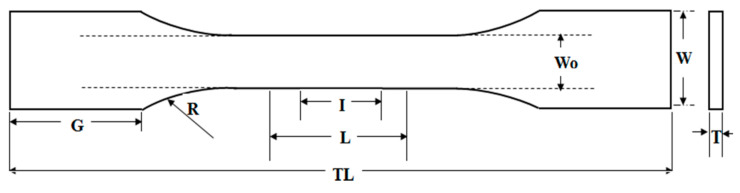
Schematic drawings of dumbell specimen according to ASTM D638 Type 5 [13].

The tensile properties such as the tensile strength, elongation at break, and Young’s modulus of the TPS-DOL biocomposite films were determined through a tensile test using the Instron Machine 5569 (Norwood, MA, USA), according to angle test specimen (ASTM D 882) [12]. The test specimens were cut into dumbbell shape according to an ASTM D 638 Type V die cutter [13]. Details are shown in Figure 3 and Table 2.

The samples were tested with a crosshead speed of 10 mm/min. Five replicates of each sample were tested, and the average values of tensile strength, elongation at break, and Young’s modulus were recorded.

### 3.4. Tear Test

**Figure 4:**
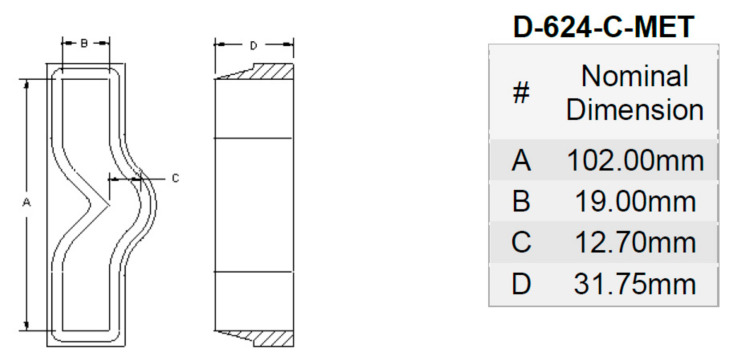
Schematic drawings of tear test specimen according to ASTM D624 Type C [14].

The tear strength of the TPS-DOL biocomposite films was determined by using an Instron Machine 5569. The samples were cut according to angle test specimen (ASTM D624 Type C) using a wood-base laser cutter [14]. Dimensions of the tear test specimen are shown in Figure 4 The test was run at a crosshead speed of 500 mm/min. Three replicates of each biocomposite film were tested and average values of tear strength were taken.

**Figure 5:**
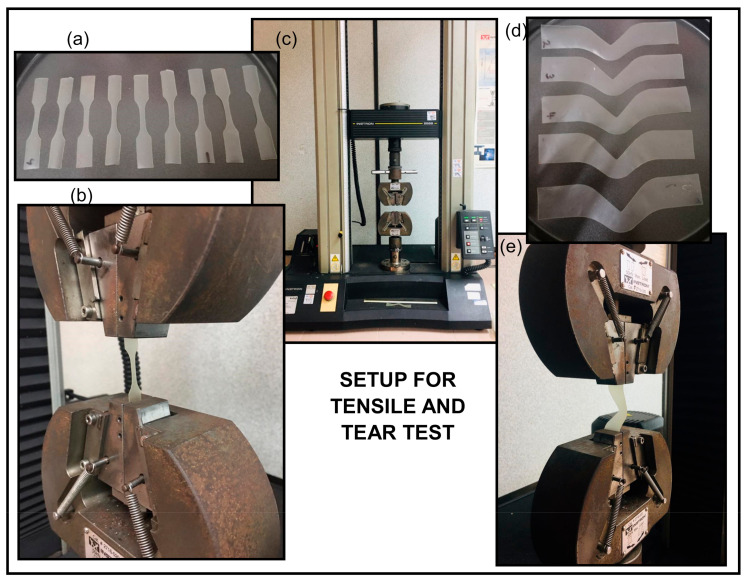
Setup for tensile and tear tests: (**a**) dumbbell-shaped samples for tensile test, (**b**) sample loading for tensile test, (**c**) Instron machine model 5569 to perform tensile and tear tests, (**d**) samples for tear test, (**e**) sample loading for tear test.

Setup for tensile and tear tests is shown in Figure 5. Both testings were carried out using the same Instron machine.

## 4. Results and Discussion

### 4.1. Functional Group Analysis of the Dolomite Filler (DOL(P) and DOL(U))

**Figure 6:**
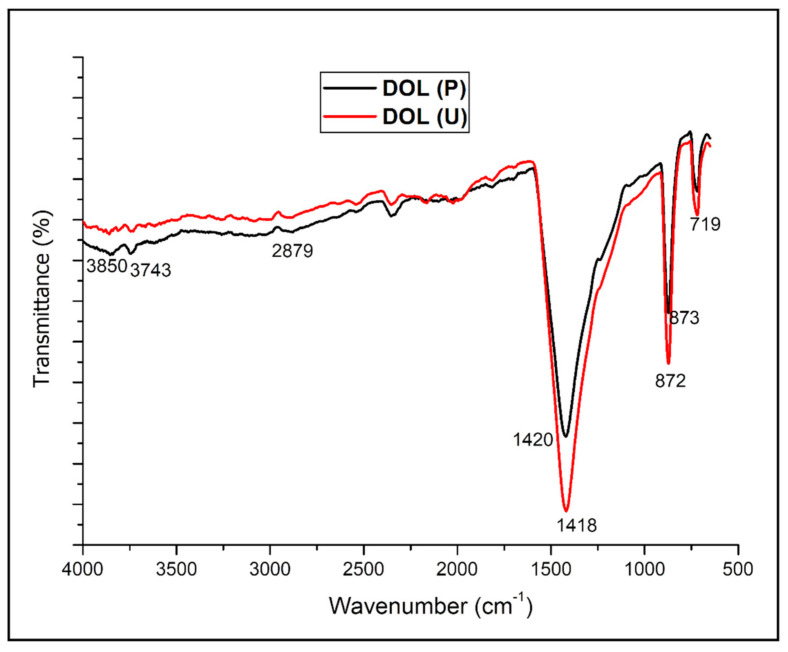
Fourier transform infrared spectroscopy (FTIR) spectra of pristine dolomite (DOL(P)) and sonicated dolomite (DOL(U)).

Fourier-transform infrared spectroscopy (FTIR) analysis was conducted for DOL(P), DOL(U), TPS, and TPS-DOL biocomposite films. Figure 6 illustrates the FTIR spectra of DOL(P) and DOL(U).

Generally, the FTIR spectra of DOL(P) indicate that the most prevalent component present in dolomite is carbonate [15]. The carbonate mineral group (magnesium and calcium carbonate) illustrates the strong peak at 1420 cm^−1^, which is associated with lattice CO_3_^2−^ [16]. On the other hand, the other main components of dolomite can be seen through the appearance of spectral peaks around 3000 and 719 cm^−1^ [15]. The FTIR absorption at around 3000 cm^−1^ relates to organic residue, while the band at around 2870 cm^−1^ represents the calcite combination band, and the peak at 719 cm^−1^ is attributed to magnesian calcite or calcite [16,17]. In agreement with our result, Chen et al. also stated that there are few FTIR absorptions at 1420, 873, and 719 cm^−1^, which are verified as the presence of dolomite [17]. DOL(U) refers to the dolomite, which has underwent a 2 h sonication process. It is observable that the features of the FTIR spectra of DOL(P) and DOL(U) are quite similar. There are only slight changes in the peak position of the overall spectra, which suggest that there is no structural and chemical modification of the dolomite when subjected to the sonication process. However, there are more intense peaks around 1418, 872, and 719 cm^−1^ for DOL(U) when compared with DOL(P). This could be due to the smaller particle size but larger surface area of dolomite after it was subjected to the sonication process. It is widely understood that the infrared (IR) spectroscopy involves the analysis on how the infrared light interacts with matter. Therefore, the concentration of molecules in the tested sample affects the peak intensity in the IR spectra [18]. Particles with higher surface area may allow penetration of the IR light into a greater number of molecules. As a result, more intense peaks can be observed. Note that the aim of conducting the sonication process in this research was to break the dolomite particles into smaller and finer size so that the dolomite filler can be more efficiently dispersed throughout the TPS matrix. This can enhance the tensile and tear properties due to better interaction between dolomite filler and the TPS matrix.

### 4.2. Functional Groups Analysis on TPS Film and TPS-DOL Biocomposite Films

**Figure 7:**
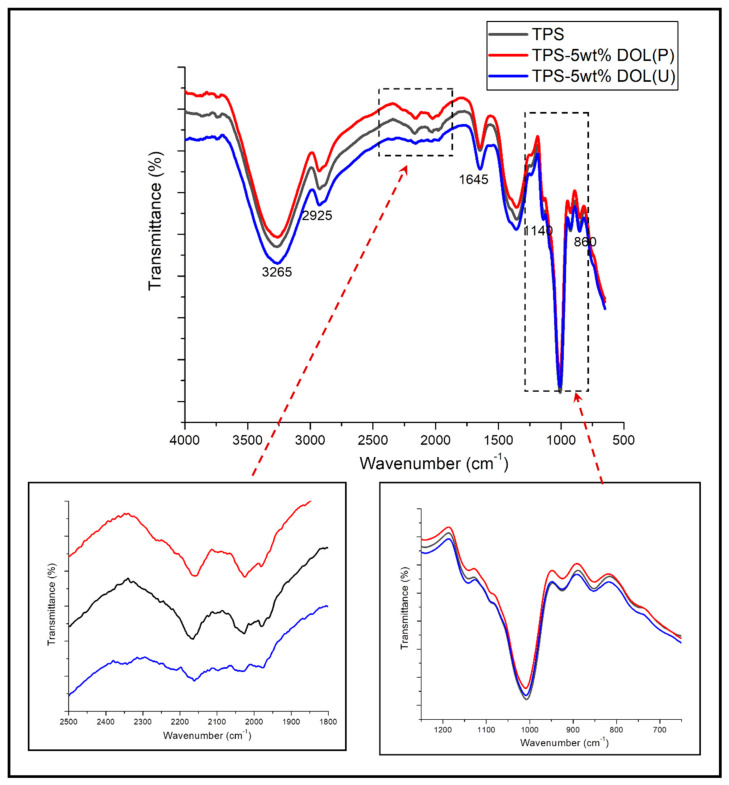
FTIR spectra of thermoplastic starch (TPS) film and TPS-5wt%DOL biocomposites films.

Figure 7 indicates the FTIR spectra of the TPS film, TPS-5wt%DOL (P) and TPS-5wt%DOL(U) biocomposite films. It can be observed that the FTIR signal of the TPS film indicates a strong spectral band in the range of 600 to 1500 cm^−1^, which confirms the fingerprint of the starch material [5]. TPS film displays the characteristics FTIR absorptions at 3265 and 2925 cm^−1^, which correspond to the O-H and C-H stretching, respectively. The appearance of the peak at 1645 cm^−1^ was due to the hydrophilic characteristics of the TPS itself, as it can be tightly bound with water [19]. The spectral peak at 1140 cm^−1^ is contributed to the C-O-H bond. Within the region below 860 cm^−1^, the spectrum of the TPS film reveals the complex vibrational mode, which was likely due to the skeletal vibrations of the pyranose ring in the glucose unit [19].

Overall, the FTIR spectra of the TPS film were almost similar to the TPS-5wt%DOL biocomposite films. Only slight changes can be observed in the range of 1800 to 2500 cm^−1^ and 750 to 1200 cm^−1^. When benchmarked with the TPS and TPS-5wt%DOL(P) biocomposite film, we can see slight broadening of the peak that relates with molecules of starch in the TPS-5wt% DOL(U) film (refer to the bands around 750 to 1200 cm^−1^). Interestingly, this was also accompanied by the broadening and weakening of peaks, which was related to dolomite (1800–2500 cm^−1^). These could be a signal that some matrix–filler interactions have occurred (perhaps hydrogen bonding) between the starch and DOL(U) filler. As mentioned earlier, the size of dolomite became much smaller upon the sonication process. Consequently, these smaller particles could be better dispersed and diffused into the TPS chains, which interact well through the formation of hydrogen bonding. Better interaction between the DOL(U) filler and TPS molecules resulted in the TPS-5wt%DOL(U) biocomposite film that possesses better mechanical properties than the TPS film, as discussed in the following section.

### 4.3. Crystallinity Analysis of the Dolomite Filler (DOL(P) and DOL(U))

**Figure 8:**
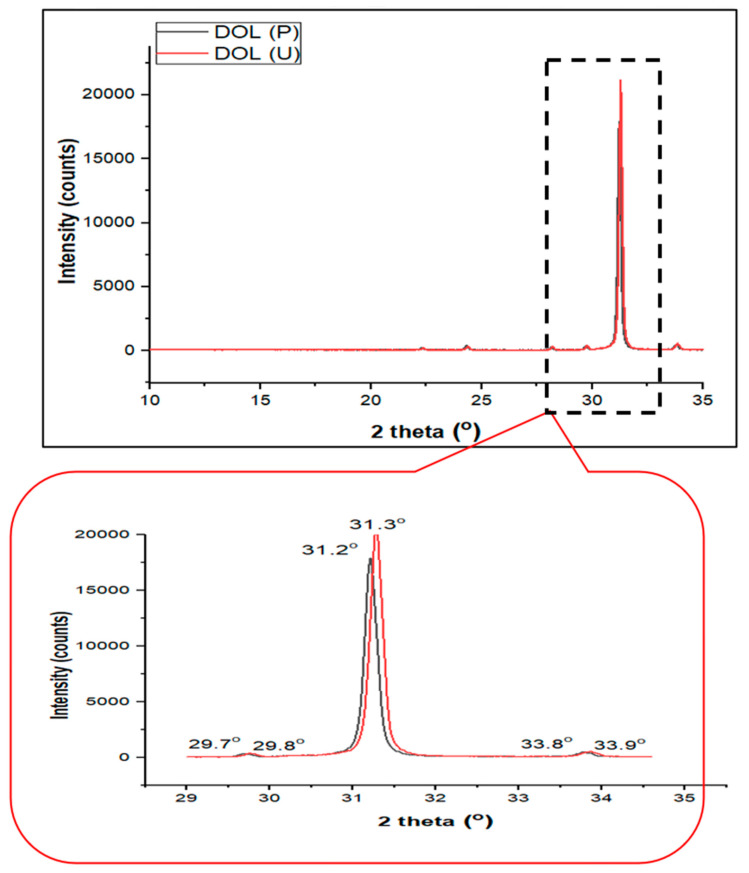
X-ray diffraction (XRD) pattern of DOL(P) and DOL(U).

The structure of dolomite is ordered; thus, its crystalline structure can be analyzed through XRD. The structure of the DOL(P) and DOL(U) was characterized and compared by using XRD analysis. An XRD pattern was used to observe the changes in the dolomite structure before and after undergoing the sonication process. Figure 8 illustrates the XRD pattern of the DOL(P) and DOL(U).

The XRD pattern of dolomite (DOL(P) and DOL(U)) reveals diffraction peaks at around 2*θ* = 31.2° and 33.8°, which are similar to that observed by Mohammed et al. [15]. However, when benchmarked with the DOL(P), the DOL(U) shows more intense diffraction peaks at both angles. This signifies that the crystallinity of dolomite was enhanced by the sonication process. To prove this, the degree of crystallinity of both types of dolomite was measured and tabulated in Table 3. The degree of crystallinity for DOL(P) and DOL(U) was found to be 76.57% and 77.10%, respectively. The higher degree of crystallinity of the DOL(U) could be as a result of the 2-h sonication process. The reduction in particle size of dolomite has increased its surface area. As a result, signals from X-ray diffraction that went through the crystalline lattices of dolomite particles became stronger [5].

### 4.4. Crystallinity Analysis of the Corn Starch, TPS Film, and TPS-DOL Biocomposite Films

XRD can be used to determine the degree of crystallinity based on the ratio of crystalline to amorphous phase in the starch. Starch is a semi-crystalline material, which consists of two main components; amylose (forming crystalline region) and amylopectin (forming amorphous phase). The existence of these two phases can be proved through the XRD spectrum.

**Figure 9:**
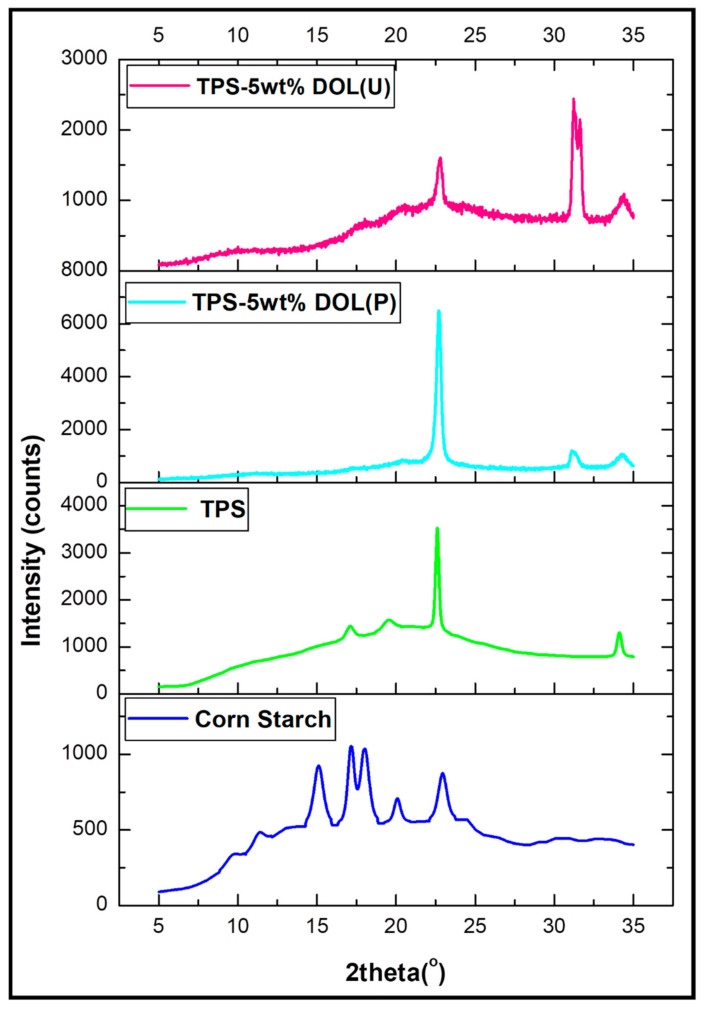
XRD pattern of corn starch, TPS film, and TPS-5wt%DOL (P) and TPS-5wt%DOL (U) biocomposite films.

The degree of crystallinity values for the corn starch, TPS film, TPS-5wt%DOL(P), and TPS-5wt%DOL(U) films were also further studied by using XRD analysis. Table 2 indicates the percent crystallinity of all the samples, while their XRD pattern in the range of 10° to 35°(2*θ*) is illustrated in Figure 9.

XRD pattern of the corn starch powder indicates strong and sharp diffraction peaks at 2*θ* = 15.1°, 20°, and 22.9° and an unresolved doublet at 17.1° and 18°, which signify a typical crystalline structure of cereal starch (A-type crystallinity). Due to the double-helical arrangement of amylopectin chains, these A-type crystallites are usually denser and less hydrated. Similar results of the pure corn starch were proved by other researchers [5,20]. The peaks at 2*θ* = 15.1° and 18° that exist in the corn starch disappeared in the XRD signal of the cast TPS film, which means the original crystalline structure was destroyed during the solution mixing process. However, there is a minimum residue of A-type crystalline that could still be observed in the TPS film produced by the solvent casting technique [21]. In the preparation of the solvent cast TPS film, shear and thermal energies applied during the stirring process were adequate to melt granular the crystallites; therefore, most of the crystalline phase of the starch has been diminished.

The XRD pattern of the TPS film has shown the combination of crystallinities Type B and Type Vh as well as the low value of the degree of crystallinity, which is about 25.38%. There are diffraction peaks at 2*θ* of 17.2°, 19.5°, 22.6°, and 34.1°. Peaks at 17.2° are assigned to B-type crystals, while peaks at 19.5° and 22.6° belong to Vh-type crystals, which are related to amylose crystallization into a single helical structure [5,22].

Upon the addition of dolomite into the starch matrix, the peak at 2*θ* = 31° (for TPS-5wt% DOL(P)) and doublet peak at 2*θ* = 31.2° and 31.6° (for TPS-5wt%DOL(U)) appear. Those peaks are correlated with dolomite, further proving the existence of dolomite particles in the structure of the biocomposite films. As referred to Table 3, it can be seen that the addition of dolomite filler significantly increased the degree of crystallinity of the TPS film. Although the peak location of starch does not change obviously in the XRD pattern, the intensities increased significantly due the addition of dolomite into the TPS film (refer to the diffraction peaks 2*θ* at around 22° for TPS film and TPS-5wt%DOL(P)) film. The degree of crystallinity of the TPS film was increased from 25.38% to 51.79% when incorporated with DOL(P).

According to Table 3, TPS-5wt%DOL(U) (52.43%) has a higher degree of crystallinity as compared with TPS-5wt%DOL(P) (51.79%). Based on Figure 9, the peak intensity for TPS-5wt%DOL(U) is higher than that of TPS-5wt%DOL(P), (refering to diffraction peaks at around 2*θ* = 31°). In addition, TPS-5wt%DOL(U) also showed a strong doublet peak at around 2*θ* at 31° due to the presence of well-dispersed dolomite particles. The de-agglomeration of dolomite upon the sonication process led to the increased surface area of the dolomite particles, thus producing a stronger diffraction peak when dispersed in the TPS matrix. Another reason could be related to the sonication process that facilitated the dispersion of dolomite particles throughout the TPS matrix and improved the matrix–filler interactions. Subsequently, this has encouraged the nucleation process for the TPS matrix crystallization and thus increased the degree of crystallinity of the biocomposites film [23]. The increase of degree of crystallinity is commonly associated with its improvement in tensile strength [5,21]. This is also the reason why the TPS-DOL(U) biocomposite film has better tensile strength when compared with TPS-DOL(P) biocomposite film, which will be shown and discussed in the next section.

### 4.5. Mechanical Properties Evaluation through Tensile Test

**Figure 10:**
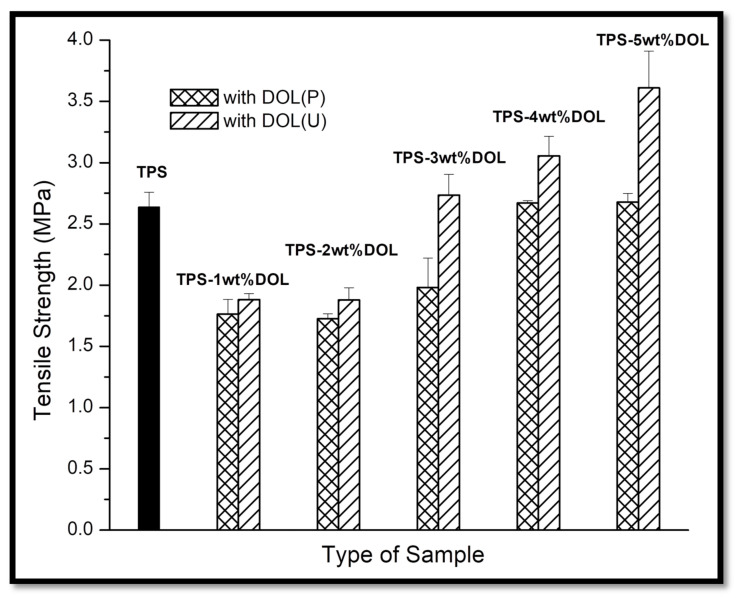
Tensile strength of TPS film and TPS-DOL biocomposite films.

**Figure 11:**
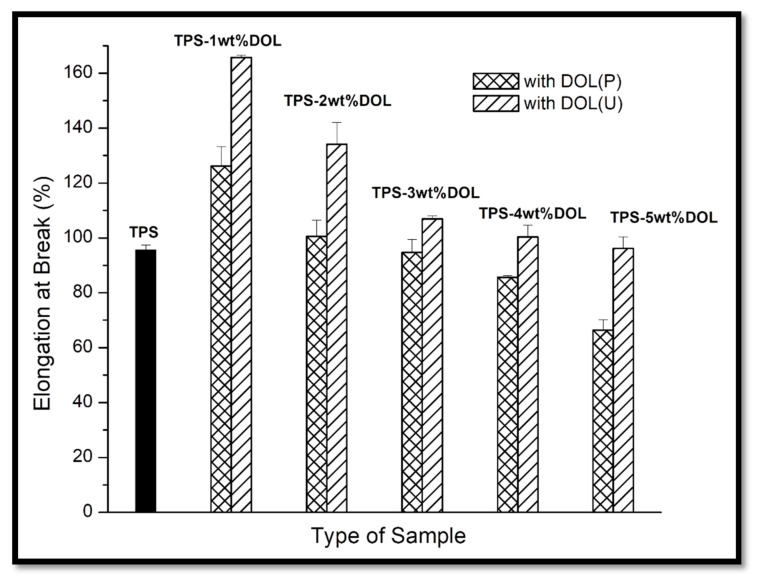
Elongation at break of TPS film and TPS-DOL biocomposite films.

**Figure 12:**
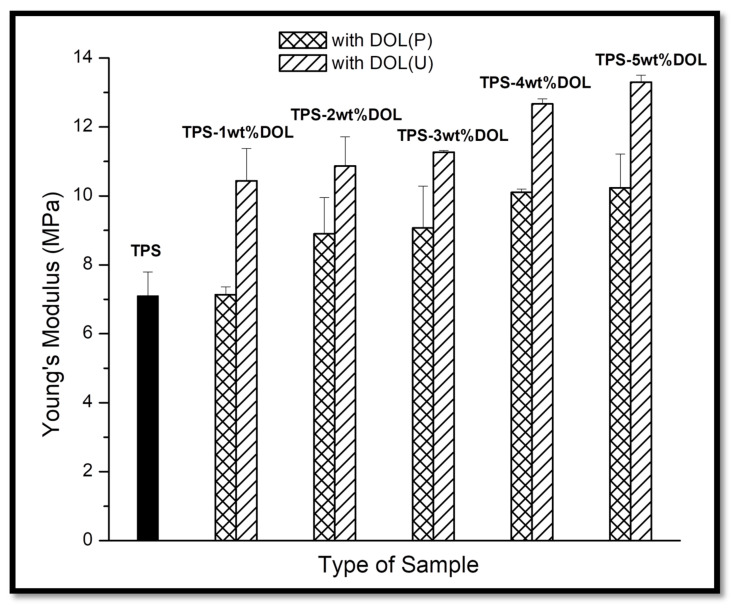
Young’s modulus of TPS film and TPS-DOL biocomposite films.

Figure 10, Figure 11 and Figure 12 illustrate the effect of dolomite loading (0, 1, 2, 3, 4 and 5 wt%) and sonication process on the tensile properties of TPS biocomposite films. According to Figure 10, low loadings of dolomite cause a deterioration of the tensile strength of the TPS-DOL biocomposite film. The TPS film possesses the tensile strength of 2.64 MPa. Meanwhile, the tensile strength of the TPS-DOL (P) biocomposites with 1, 2 and 3 wt% filler was found to be 1.76 MPa, 1.73 MPa, and 1.98 MPa, respectively. On the other hand, TPS-DOL(P) biocomposite films with high loading of filler (4 and 5 wt%) show almost the same tensile strength value with the TPS film. This assessment was made based on the tensile strength and standard deviation values of the TPS film and TPS-DOL(P) biocomposite films containing 4 and 5 wt% DOL(P), which show statistically no significant difference. The results suggest that dolomite in pristine form was not a good reinforcing filler for the TPS film and thus could not improve the tensile strength of the host biopolymer when added in 1 to 5 wt%. Conversely, for TPS-DOL(U) biocomposite film cases, the tensile strength was enhanced when DOL(U) was added in 4 wt% and 5 wt%. Based on the results, the tensile strength of TPS-4wt%DOL(U) biocomposites was 3.06 MPa, which showed an improvement of about 15.9% when compared with the TPS film. However, when less DOL(U) was added into the TPS (1 wt%, and 2 wt%), the tensile strength was 1.88 MPa and 1.87 MPa, respectively, which is somewhat lower than the TPS film. The TPS-5wt% DOL(U) biocomposite has the highest tensile strength among all the samples (3.61 MPa) by demonstrating 36.74% higher in tensile strength when benchmarked with the TPS film.

Overall, the results indicate that the low loading of dolomites, either in pristine or sonicated form, could not improve the tensile strength of the TPS biocomposite film. This could be due to the insufficient content of dolomite particles to allow a reinforcing effect to the TPS chains. This happened due to the lack of dispersed particles to act as a stress-transferring medium when subjected to the tensile load. In fact, the lowering of tensile strength can be seen due to the presence of filler that induces inhomogeneity in the biocomposite system, rather than providing reinforcing effect. Syed Adam et al. have obtained and reported the same findings through their research on TPS biocomposite [7]. However, when a higher content of dolomite was added, there was a greater number of particles being diffused into the TPS chain and acting as reinforcing filler. Upon the sonication process, the dolomite filler turned into smaller size particles, which can be more easily dispersed throughout the TPS matrix. There were higher numbers of well-dispersed dolomite particles to interact with TPS molecular chains. As observed through the FTIR analysis, such interactions can be observed through the formation of hydrogen bonding between the dolomite filler and TPS molecular chains. As a result, a better stress-transforming mechanism between the matrix and filler occurred. Apparently, the tensile strength of the TPS-5wt%DOL(U) biocomposite film (3.61 MPa) was better than the tensile strength of the TPS-5wt%DOL(P) biocomposite film (2.68 MPa). This shows that the sonication process can assist the distribution and dispersion of dolomite filler throughout the TPS, which eventually improved the tensile strength of the biocomposite film. A better dispersion of dolomite could increase the efficiency of the filler to transfer the stress effectively within the composite structure. This is in agreement with the findings of Nik Adik et al. [24].

Elongation at break values of the TPS film, TPS-DOL(P) biocomposite, and TPS-DOL(U) biocomposite films are compared in Figure 11. It can be seen that as the DOL(P) and DOL(U) loading increased in the TPS matrix, reduction in the elongation at break occurred. Elongation at break is a reflection of the ductility of the material: the direct opposite for brittleness. When dolomite filler is incorporated into a TPS matrix, it will increase its stiffness. The increase in stiffness resulted in a decrease in the ductility of the material. Thus, as the dolomite loading increases, the ductility decreases. Such a trend was also reported by other researchers [24,25]. The elongation at break of the TPS film was found to be 95.6%. With the addition of 1 wt% DOL(P) in TPS film, the elongation at break was 126.13%. The elongation at break was reduced significantly (25.43%) when the DOL(P) loading was increased to 2 wt%. When the loading of DOL (P) increased to 3 wt% and 4 wt%, the elongation at break value continued to decrease to 94.7% and 85.67%, respectively. TPS-5wt%DOL(P) has the lowest elongation at break among all the samples (66.37%), showing 30.58% reduction when benchmarked with the TPS film. Apparently, TPS filled with DOL(U) has a similar trend with TPS filled with DOL(P). The elongation at break of TPS-1wt%DOL(U) biocomposites was 165.77%, but it reduced significantly by 23.59% to 134.13% when the loading of DOL(U) was increased to 2 wt%. A further increase of DOL(U) loading to 3 wt% and 4 wt% resulted in the decrease of elongation at break to 106.9% and 100.37%, respectively. The TPS-DOL(U) biocomposite film achieved the lowest elongation at break (96.17%) when the filler loading was 5 wt%. As expected, the elongation at break value of the TPS-DOL(P) biocomposite films is lower than the TPS-DOL(U) biocomposite films when the same amount of filler was added. For instance, the elongation at break of TPS-1wt%DOL(P) (126.13%) was lower compared with TPS-1wt%DOL(U) (165.77%). This was probably due to the greater aggregation of dolomite filler when no sonication process was involved, thereby resulting in a greater stiffening effect to the TPS molecular chains. Subsequently, the flexibility of the biocomposite film reduced more significantly.

Young’s modulus values of the TPS film, TPS-DOL(P), and TPS-DOL(U) biocomposite films at different dolomite content (1 wt%, 2 wt%, 3 wt%, 4 wt%, and 5 wt%) are demonstrated in Figure 12. It can be seen that the addition of DOL(P) and DOL(U) into the TPS matrix resulted in the increase of the Young’s modulus of the biocomposites. In general, the Young’s modulus of these samples was enhanced by the incorporation of dolomite filler in the TPS matrix. This was due to the stiffness of dolomite, which can restrict the chain mobility of the TPS matrix. The results are in tandem with findings reported by a few researchers [24,25,26]. It can be concluded that the TPS film has the lowest Young’s modulus (7.1 MPa) among all the samples. The Young’s modulus of TPS-5wt%DOL(P) biocomposite film was 10.23 MPa, which possesses a 44.08% increment when benchmarked with the TPS film. The TPS-5wt%DOL(U) biocomposite achieved the highest Young’s modulus (which represents the stiffest samples) among all the samples. It possesses an 87.32% increment in Young’s modulus when benchmarked with the TPS film. Moreover, the value of Young’s modulus obtained for TPS-DOL(U) biocomposite films was higher than that of TPS-DOL(P) biocomposite films. The most significant difference can be observed when incorporating 5 wt% of dolomite into the TPS matrix. The TPS-5wt%DOL(U) (13.3 MPa) achieved a 30% increment in Young’s modulus when compared with TPS-5wt%DOL(P) (10.23 MPa). As explained previously, dolomite that underwent a sonication process can be more homogeneously dispersed throughout the TPS matrix. Better filler–matrix interactions cause more restriction in TPS chain mobility and thus increase the Young’s modulus of the biocomposite film [5,7].

### 4.6. Mechanical Properties Evaluation through Tear Test

**Figure 13:**
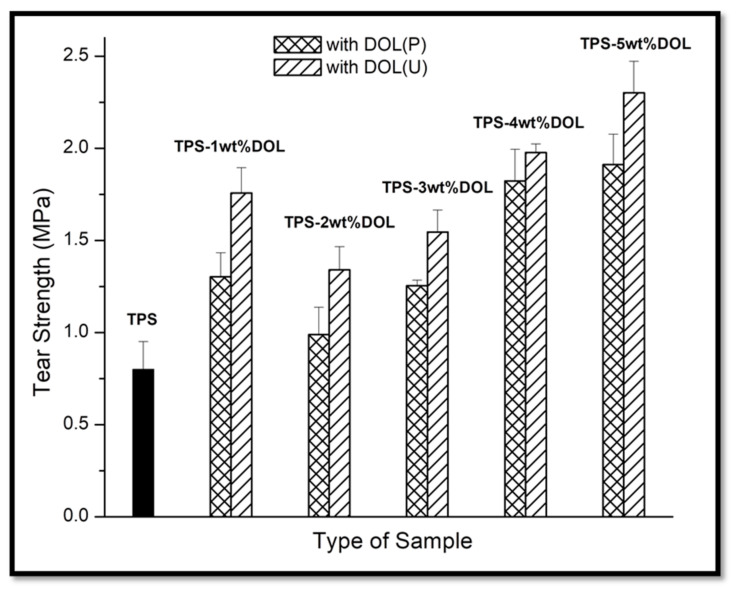
Tear strength of TPS film and TPS-DOL biocomposite films.

Figure 13 compares the values of the tear strength of the TPS film and TPS-DOL biocomposite films. Generally, the tear strength of the TPS film (0.80 MPa) is lower than that of the TPS-DOL(P) and TPS-DOL(U) biocomposite films. This indicates that both pristine dolomite and sonicated dolomite have successfully improved the tear strength of the TPS. Both TPS-DOL(P) and TPS-DOL(U) biocomposite filmss achieved the highest tear strength when 5 wt% of filler was employed. TPS-5wt%DOL(U) biocomposite film shows the highest tear strength (2.30 MPa) among all the samples. It has achieved 187.50% higher tear strength when compared with the TPS film.

Obviously, when the same amount of filler was used, the TPS-DOL(U) biocomposite film possesses greater tear strength as compared to the TPS-DOL(P) biocomposite film. This happened due to the sonication process that helps to homogenously distribute dolomite particles into TPS molecule chains and improve the matrix–filler interactions [5]. The homogenous distribution of dolomite can increase the efficiency of filler to transfer the load within the TPS biocomposite structure. Obviously, the tear strength data of the biocomposite seem to follow the trend of the tensile strength data, in which the higher loading of dolomite filler causes a better reinforcing effect. The following statement explains this phenomenon: When a greater amount of dolomite particles penetrated through the polymer chain, they would develop more surface interactions with the TPS matrix; therefore, a better reinforcing effect can be seen. This is translated through the tensile and tear strength values. Osman, Edwards, and Martin have observed the same phenomenon in their composite system with mineral filler (clay) [27].

### 4.7. Morphology of Tensile Fractured Surface of the TPS Film and TPS-DOL Biocomposites

**Figure 14:**
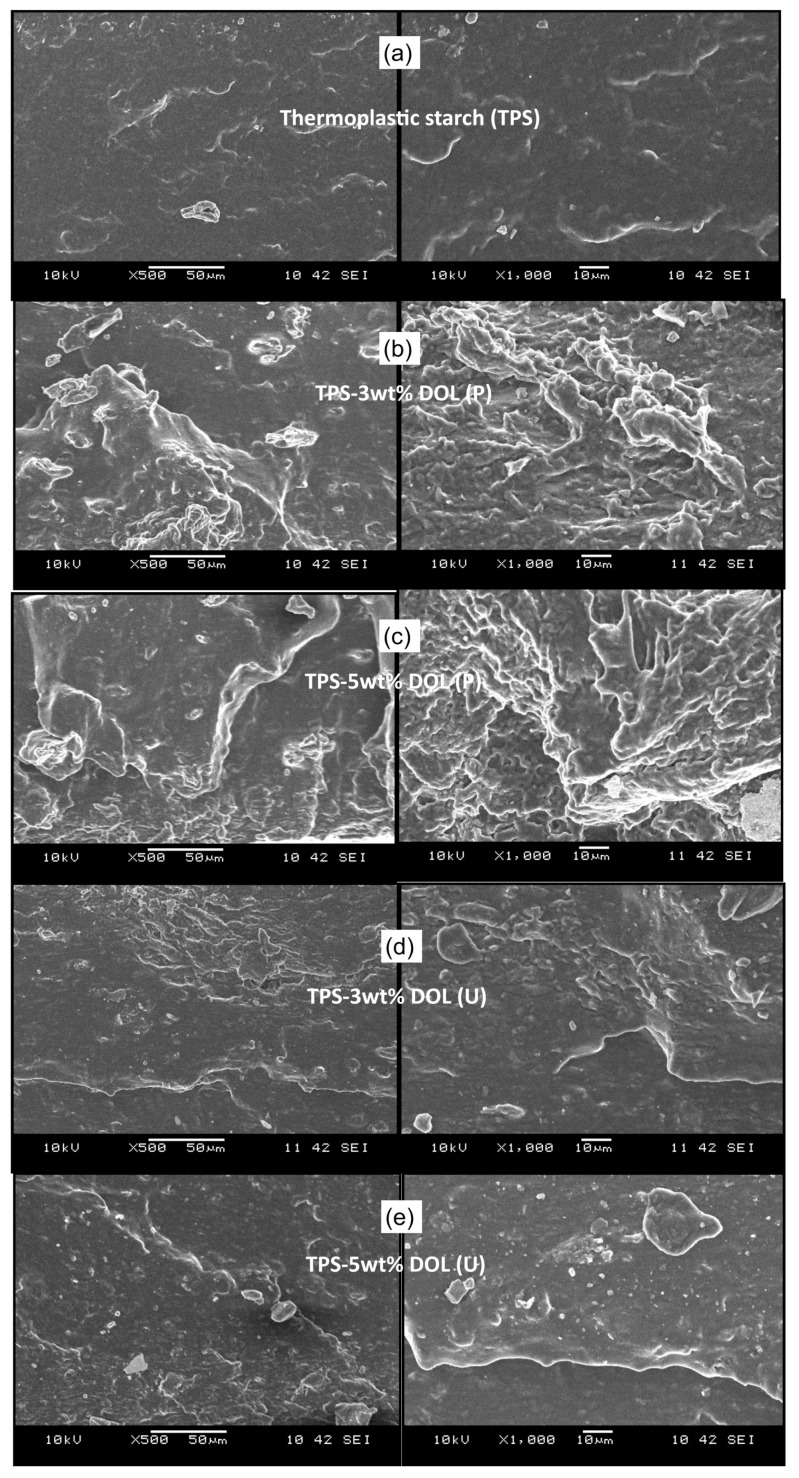
SEM images of tensile fractured surface of the (**a**) TPS film (**b**) TPS-3wt%DOL (P) (**c**) TPS-3wt%DOL (U) (**d**) TPS-5wt% DOL (P) and (**e**) TPS-5wt%DOL (U) biocomposite films at 500× magnification (left) and 1000× magnification (right).

Figure 14 presents the SEM images of the tensile fractured surface of the TPS film and TPS-DOL biocomposite films. In its neat form (without filler), the TPS film shows a smooth and homogeneous surface. However, upon the addition of pristine dolomite (DOL(P)), the surface morphology of the matrix became rough and non-homogeneous with pores and pits. Apparently, at the same dolomite loading, the TPS-DOL biocomposites with sonicated filler (TPS-DOL(U)) exhibit smoother and more homogeneous surface morphology than the TPS-DOL biocomposites with pristine dolomite (TPS-DOL(P). These findings support the results of a test where the addition of DOL(U) in the TPS resulted in a more homogeneous biocomposite due to the well-dispersed filler distributed in the matrix. As a result, the tensile properties of the TPS film were enhanced. Li et al. have observed similar features in their poly (3-hydroxybutyrate-co-3-hydroxyvalerate) (PHBV)-based biocomposites with miscanthus biocarbon as the filler. A homogeneous dispersion of filler has been found to improve the mechanical and thermal stability of the host biopolymer. The biocomposite with a poor dispersion of filler indicated a rough fractured surface with the appearance of voids, suggesting the poor wetting of filler by the biopolymer matrix [28].

## 5. Summary

The effects of dolomite loading and the sonication process on the mechanical properties of the TPS-DOL biocomposite films have been explored through this research. Indicatively, the use of high loading of dolomite (4 to 5 wt%) can improve both the tensile and tear properties of the biocomposite film, provided that the dolomite is being sonicated prior to being added into the TPS matrix. The use of dolomite in pristine form (DOL(P)) could not improve the tensile properties, even when added in 5 wt%. Conversely, upon the sonication process, the dolomite (DOL(U) brought an increment in both tensile and tear strength of the biocomposite film when added in 4 and 5 wt%. Based on the FTIR results, the spectral peaks of DOL(U) were more intense when compared with DOL(P). This means that when dolomite underwent the sonication process, a reduction in particle size occurred, causing a larger surface area of particles for the penetration of IR light into a greater number of molecules. In addition, the sonication process also reduced the particle size of the dolomite, assisting the dispersion and distribution of its particles throughout the TPS matrix. XRD analysis showed that the sonication process has increased the crystallinity of dolomite. This factor also contributed to the enhancement of the tensile and tear properties of the biocomposite film. The fractured surface morphology of the TPS-DOL (U) biocomposites is smoother and more homogeneous than the TPS-DOL (P) counterparts, further supporting the fact that the sonication process can improve the dispersion of the dolomite filler throughout the TPS matrix and produce more homogeneous biocomposites. In conclusion, sonicated dolomite has potential to be used as a reinforcing filler in the TPS film in order to improve its viability for packaging application. The low cost and abundancy of dolomite and the simple and environmental friendly method of sonication can be adopted to produce a sustainable biocomposite for future needs.

## Figures and Tables

**Table 1:** The formulation of thermoplastic starch-dolomite (TPS-DOL) biocomposite films with their respective acronyms.

TPS (wt%)	Dolomite Filler without Sonication Process (wt%)	Dolomite Filler Subjected to 120 min Sonication Process (wt%)	Acronym of Biocomposite Film
100	-	-	TPS
99	1	-	TPS-1wt%DOL(P)
99	-	1	TPS-1wt%DOL(U)
98	2	-	TPS-2wt%DOL(P)
98	-	2	TPS-2wt%DOL(U)
97	3	-	TPS-3wt%DOL(P)
97	-	3	TPS-3wt%DOL(U)
96	4	-	TPS-4wt%DOL(P)
96	-	4	TPS-4wt%DOL(U)
95	5	-	TPS-5wt%DOL(P)
95	-	5	TPS-5wt%DOL(U)

**Table 2:** Dimension of a dumbbell specimen (ASTM D638 Type V).

Symbol	Description	Dimension (mm)
I	Gauge length	7.62
L	Length of narrow selection	9.53
Wo	Width of narrow selection	3.18
G	Grip length	19.05
W	Total width	9.53
TL	Total length	63.50
R	Radius of curvature	12.70

**Table 3:** Crystallinity of the pristine dolomite (DOL(P)), sonicated dolomite (DOL(U)), corn starch, TPS film, TPS-5wt%DOL (P), and TPS-5wt%DOL (U) biocomposite films based on XRD analysis.

Samples	2*θ* (°)	Percent (%)	
		Crystallinity	Amorphous
DOL(P)	22.4, 24.4, 28.2, 29.7, 31.2, 33.8	76.57	23.43
DOL(U)	22.4, 24.3, 28.1, 29.8, 31.3, 33.9	77.10	22.90
Corn starch powder	11.4, 15.1, 17.1,18, 20, 22.9	58.03	41.97
TPS	17.2, 19.5, 22.6, 34.1	25.38	74.62
TPS-5wt%DOL (P)	20.4, 22.7, 31, 34.3	51.79	48.21
TPS-5wt%DOL (U)	17.4, 20.2, 22.8, 31.2, 31.6, 34.4	52.43	47.57

## Data Availability

The data presented in this study are available on request from the corresponding author.

## Abbreviations

ASTM D	American Society for Testing and Materials D Standard
DOL (P)	Pristine dolomite
DOL (U)	Sonicated dolomite
FTIR	Fourier Transform Infrared Spectroscopy
ISO	International Organization for Standardization
mm	Micronmeter
MPa	Mega Pascal
PE	Polyethylene
PP	Polypropylene
TPS	Thermoplastic starch
TPS-Xwt%DOL(P)	TPS biocomposite containing X wt% pristine dolomite
TPS-Xwt%DOL(U)	TPS biocomposite containing X wt% sonicated dolomite
XRD	X-ray Diffraction
μm	Micrometer
